# Attention and impulsivity assessment using virtual reality games

**DOI:** 10.1038/s41598-023-40455-4

**Published:** 2023-08-22

**Authors:** David Mendez-Encinas, Aaron Sujar, Sofia Bayona, David Delgado-Gomez

**Affiliations:** 1 Departamento de Estádistica, Universidad Carlos III de Madrid, Leganes, Spain; 2 Departamento de Ciencias de la Computación, Universidad Rey Juan Carlos, Móstoles, Spain

**Keywords:** Psychology, Human behaviour

## Abstract

The assessment of cognitive functions is mainly based on standardized neuropsychological tests, widely used in various fields such as personnel recruitment, education, or health. This paper presents a virtual reality game that allows collecting continuous measurements of both the performance and behaviour of the subject in an immersive, controllable, and naturalistic experience. The application registers variables related to the user’s eye movements through the use of virtual reality goggles, as well as variables of the game performance. We study how virtual reality can provide data to help predict scores on the Attention Control Scale Test and the Barratt Impulsiveness Scale. We design the application and test it with a pilot group. We build a random forest regressor model to predict the attention and impulsivity scales’ total score. When evaluating the performance of the model, we obtain a positive correlation with attention (0.434) and with impulsivity (0.382). In addition, our model identified that the most significant variables are the time spent looking at the target or at distractors, the eye movements variability, the number of blinks and the pupil dilation in both attention and impulsivity. Our results are consistent with previous results in the literature showing that it is possible to use data collected in virtual reality to predict the degree of attention and impulsivity.

## Introduction

One of the main current problems in child psychiatry is obtaining an accurate diagnosis of attention-deficit/hyperactivity disorder (ADHD). It has been shown that medical professionals commit about 20% false positives and 20% false negatives^1^. Many of the games focused on ADHD diagnosis are developed targeting children and adolescents, so there remains a need to develop therapeutic tests for adults^2^. In addition, high impulsivity and inattention can also occur in other mental disorders and conditions. For example, high impulsivity is associated with suicidal ideation^3^ or addictions^4^. On the other hand, inattention is a factor present in schizophrenia, anxiety, depression, post-traumatic stress disorder, epilepsy, among others^5^. The importance of being able to evaluate these two constructs is not restricted only to the health field. Several studies have shown their relationship with academic performance^6,7^. In other fields, such as personnel recruitment, inattention or impulsivity are characteristics that are taken into account in the selection of workers such as air traffic controllers or policemen^8,9^. All these facts illustrate the importance of obtaining accurate assessments of a person’s impulsivity and inattention.

Inattention and impulsivity are frequently assessed using the completion of scales and questionnaires. It is frequently conducted using the completion of scales and questionnaires. However, some studies point out the disadvantages of these methods, such as subjective interpretation, lack of precision^10^, or malingering to obtain a benefit (economic, academic, access to drugs, etc.)^11^.

An alternative to the scales and questionnaires is the continuous performance tasks (CPT), originally created to prove that brain-damaged patients performed tasks worse than healthy controls^12^. These evaluations typically consist of the participant trying to distinguish between target/non-target stimuli in order to conduct a given action, more commonly known as the go/no-go paradigm^13^. These stimuli are presented on a computer so that reaction time and errors can be measured without human supervision.

Recently, with the advances in computer science, the trend is towards the creation of more advanced assessments through video games to support diagnosis and cognitive assessment^14^. In general, these games are mostly, but not exclusively, oriented to child and adolescent related disorders^15,16^. By appearing more closely related to everyday-life hobbies and offering pleasant and fun environments, they improve participation and achieve a more realistic performance^17^. If necessary, the game and tasks can often be adapted to adults by modifying the appearance and mechanics for a more mature version^18^. On the one hand, many of the video games proposed are gamified versions of CPTs^19,20^ or versions based on the go/no-go paradigm seeking a direct comparison with an already established instrument^21,22^. On the other hand, assessment can also be done with other types of games that mimic the look and feel of other commercial games, providing a more natural and familiar experience^23,24^.

In this paper, we present an approach to support cognitive assessment using a virtual reality (VR) application that tries to predict the degree of attention and impulsivity of the participant. The application gathers variables related to the user’s movements through the use of VR goggles and in-game performance. Variables are recorded in a continuous manner during the entire time the player is in the game. Our hypothesis is that applying machine learning techniques to these variables will allow us to predict the degree of impulsivity and attention of the participant.

The remaining of the article is structured as follows. In the next section, we will cover some of the most relevant psychometrical tests, starting with self-report scales and evolving to VR and video game-based assessments. Then, in Section “The treasure hunt VR application”, we will describe in detail the proposed VR application, the registered variables and the analytical methods applied. Section “Experimental results” describes the experiment conducted on the model’s performance and the results obtained. Finally, we will discuss the implications and limitations of our results in Section “Discussion”, and conclude and propose future work in Section “Conclusion”.

## Related work

Attention and impulsiveness are frequently measured through self-reporting scales. A self-report scale widely used to measure attention control^25^ is the Attention Control Scale (ATTC) questionnaire^26^ . In the case of impulsivity, the Barratt Impulsiveness Scale (BIS-11) is one of the most commonly administered self-report scales for the assessment of impulsiveness in both research and clinical settings^27^. In this work, we will use our VR application to predict the scores of these two well-validated self-report scales.

Other approaches, instead of relying on questionnaires, focus on performing tasks. An example of this is the d2 Test of Attention^28^ which measures attention and concentration processes. The task consists of detecting target characters (the character ’d’ with two marks above it, around it or below it) that are interspersed with non-target characters (character ’p’ or character ’d’ with more or less than two marks). The Familiar Figures Matching Test (MFF20)^29^ is a matching task where the participant must select a target, from a set of six perceptually similar pictures, that exactly matches the sample. This test is able to discriminate reflective from impulsive children.

With the advance of technology, computerized tests emerged in which, for example, a series of visual and/or auditory stimuli are presented over a period of time. Those tests allow all subjects to be exposed to the same tasks and stimuli while recording variables automatically. The CPTs have been used in a variety of settings, for instance, to test the attention of brain-damaged patients^12^, or to test the skills of radar operators^30^. Two of the most commonly used CPTs are the Conners CPT^31^ and the Test of Variables of Attention (TOVA)^32^. These tests require the participant to maintain attention on a changing stimulus displayed on the screen, and they must react when a target stimulus appears. However, they differ on some key points^33^, where Conners CPT uses a “response inhibition” paradigm (90% target and 10% non-target), TOVA uses a mix of this and a “rare target” paradigm (22.5% targets and 77.5% non-targets). Furthermore, TOVA is not based on letters, but uses the positions of a square (up and down) as the target stimuli. In this way, no linguistic demands or left-right discrimination are required.

With the aim of finding new predictors, other authors have incorporated new technologies into computerized tests to take into account other variables like the patient’s movement. For example, O’Mahony et al.^34^ used wrist and ankle trackers while performing the TOVA test. Delgado-Gomez et al.^35^ adapted the CPT using the Microsoft Kinect by replacing key presses with arm movements. Valera Casal et al.^36^ found that eye vergence can be used to discriminate attention-deficit/hyperactivity disorder (ADHD) in children. Snyder et al.^37^ affirm that electroencephalographic (EEG)-based assessment obtained while participants conduct CPTs can be used to improve the accuracy of ADHD diagnosis.

Some works included the CPT in VR applications. The Virtual Classroom^20^ is a VR solution that introduces users into a classroom to perform a CPT on the blackboard. Rodriguez et al.^38^ found that Aula Nesplora, another VR-CPT solution within a virtual class, is a better predictor of ADHD than TOVA.

In order to improve participation in the assessment process and increase their ecological validity^39^, serious games and gamification have been exploited to enhance existing CPTs or to create new assessment alternatives. For example, MOXO-CPT^19^ uses pictures instead of letters. In comparison to the use of letters that are clearly distinctive target/non-target stimuli, similar pictures that share shapes and colours without being entirely equal are used. Also, it incorporates auditory and visual distractors as external interfering stimuli.

Other solutions more similar to commercial video games have also been developed for assessment or treatment. One of the most known examples is Akili’s EndeavourRx^22^ which was the first therapeutic game cleared by the U.S. Food and Drug Administration (FDA) intended to relieve and treat ADHD. The game Antonyms^21^ contains different activities within a storyline, where each activity is inspired by classic neuropsychological tasks and players have to fill a backpack or move along a route based on the go/no-go paradigm. Timo’s Adventure^40^ is another computer-based game, which through a storyline with different mini-games, is able to measure executive functions (EFs) like attention or inhibition. Delgado-Gómez et al.^23^ developed an infinite runner-based computer game, where a raccoon has to jump between platforms to reach the end, to assess attention capacity in ADHD patients. The authors found a link between the jump distance and omissions with the Strengths and Weaknesses of ADHD-Symptoms and Normal-Behavior (SWAN) scale^41^.

Our proposal is based on creating a VR application that includes a game that allows continuous data collection. We record information such as errors or reaction time and the VR device allows us to obtain eye movement information. We then use all these data to create a model to predict the scores obtained on the ATTC and BIS-11 tests, related to attention and impulsivity.

## The treasure hunt VR application

In this section, we will explain in detail the VR application developed. The application is composed of two modules. The first consists of the game itself, which allows for the collection of the variables related to the user’s performance and behaviour. The second module implements the design of the analytical model to predict the degree of attention and impulsivity.

**Figure 1:**
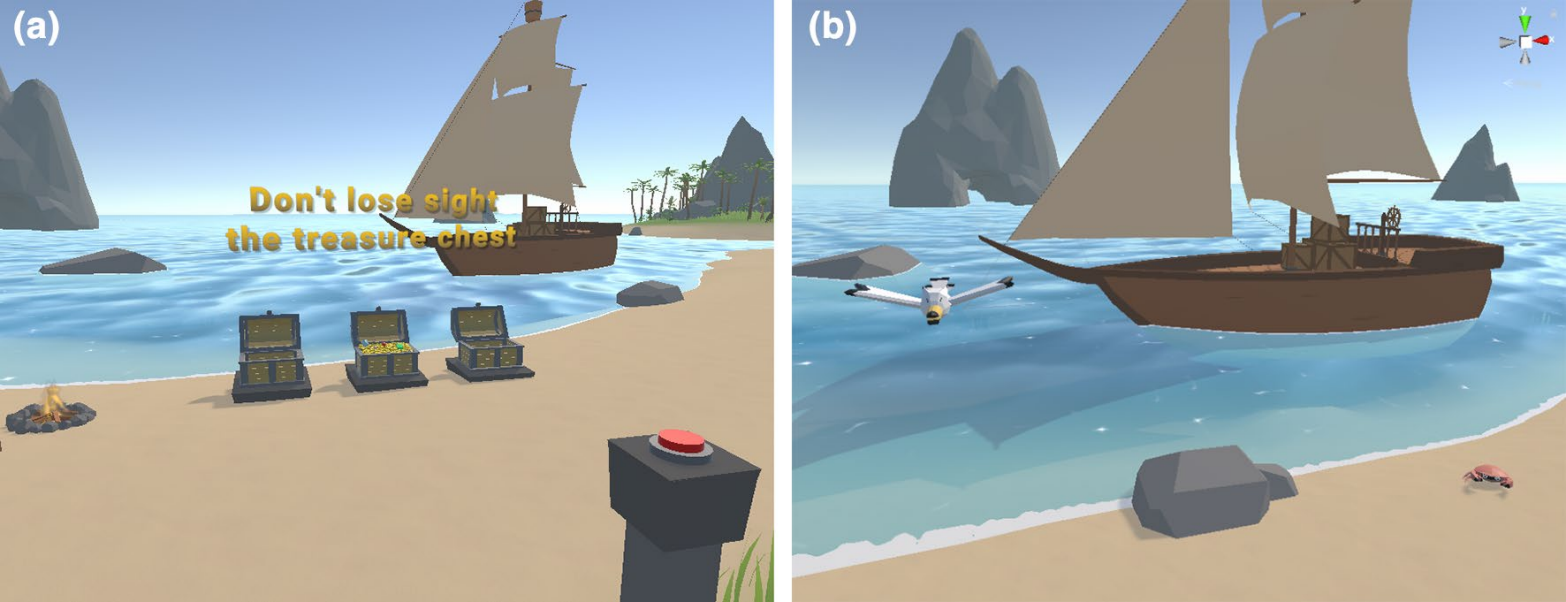
Image of the game’s beach scenario.

### Game description

The game for the VR application consists of performing a sustained attention task where distractors appear and the user must resist the impulse of diverting the gaze in order not to lose sight of the target.

The setting of the game is an island. On the sandy seashore, three treasure chests are lined up. Two of these chests are empty, while inside the other chest, the player can see that there is a treasure (see Fig. 1). The chests close and begin to alternate their position with each other, similarly to the three shells and a pea game. Players must keep their focus on the closed chest containing the treasure to be able to select it once the movement of the chests ceases. Once selected, the chests are opened and players can see if they made the right choice. After some time, the chests are closed again, starting a new round of movement. Between rounds, there is a fixed time when the chests will not move to create the need to keep watching them until they close and start moving again.

The sequence of movements of each chest is fixed and will be common to all players. In addition, from time to time distractors are introduced into the scene, such as seagulls flying or crabs moving. The complete game is made up of twenty sequences in which both the number of movements of the chests and the speed of the movements vary (it can be found as Supplementary Fig. S1 online). The total duration of the entire test is about ten minutes. This time varies slightly depending on how long each participant took to choose the chest, as we allowed a maximum of ten seconds for each selection.

The application requires a VR headset and hand controllers to gather the following variables during the game playing time: eye gaze (left/right eye 3D vector), pupil dilation, blinks, eye vergence, and hand movement. Throughout the execution of the game, the application stores the player’s performance through the following variables: the reaction time, the time spent looking at the correct chest, looking at a wrong chest or at each distractor, the number of successes, the number of errors, (either because no chest was selected within the 10 seconds or because a wrong chest was selected).

### Analytical methods

In order to find out how the variables collected by our application can help to predict the scores of the scales administered, we use a regression analysis. The chosen machine learning technique was random forest because it works well with high-dimensional data and the interpretation of the model variables is easier to understand than in other machine learning techniques. Concisely, the Random Forest technique constructs several decision trees from randomly obtained subsets of the available observations and predictors. Once the Random Forest is constructed, it classifies each new observation by averaging the predictions made by each of these trees^42^.

## Experimental results

In this section, we describe the experiment developed to evaluate the performance of the proposed application. We start by explaining the sample, the set-up and then, we detail the results obtained.

### Experiment description

We run a pilot study to evaluate our analytical model. We recruited students from the Carlos III University and the IES Jose Hierro High School, Spain. The sample was composed of 34 students with ages between 15 and 26 (mean = 19.24, SD = 2.985 and 37% of them were women). None of them had vision problems or epileptic disorders.

At the beginning, students were informed that their participation was voluntary and under no circumstances was it considered in their academic evaluation. Subjects had the possibility to withdraw from the experiment at any time if they felt some kind of sickness derived from VR or for no reason. An identification number was assigned to each participant to anonymize the data. All participants were informed of the study and signed the required informed consent form.

**Figure 2:**
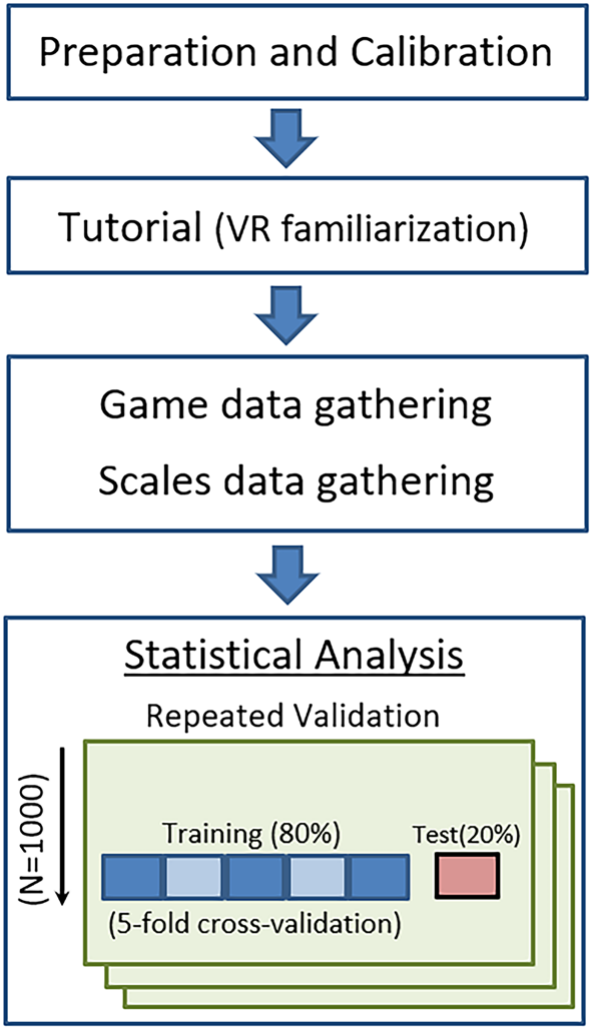
Summary of the assessment process using machine learning.

Fig. 2 shows the different steps that compose the following experiment. Participants were assisted to put on the headset ensuring a good position and the calibration process was started. The players were given the hand controls. Before playing the game, participants went through a practical tutorial in which the basic VR controls were explained so that they would be comfortable with the VR experience. After this familiarization, participants played the game. During the game, the previously mentioned variables were collected by the VR headset at a rate of 90 times per second. However, we observed that several occlusion errors had been produced when registering the hand movements, so we discarded this information for our model.

After the game is over, and in order to determine how well our application is capable of predicting the degree of attention and impulsivity, we administered the Attention Control Scale (ATTC) questionnaire^26^ and the Barratt Impulsiveness Scale (BIS-11)^27^. The first scale is composed of 20 items that are assessed on a four-point Likert scale from 1 (rarely) to 4 (always). The BIS-11 scale is designed to assess the impulsivity of a person. It has 30 items that are also assessed on a four-point Likert scale with some reverse scoring. On average, the whole procedure (familiarization, playing the game and filling the questionnaires) lasted about 20 minutes per user.

As explained, the VR and game related variables, together with the answers to the questionnaires were used to evaluate the performance of our application using a random forest in a repeated validation set-up. For each repetition, the sample is divided into 80% train and 20% test. In addition, a five-fold cross-validation was conducted on the training data to obtain the best parameters of the random forest model in each repetition. The number of repetitions was set to 1000 times. The score metrics used to measure the model performance were the Root Mean Squared Error (RMSE) and the correlation.

In our work, we used the HP Reverb G2 Omnicept goggles with its two-hand motion controllers. The main reason for this choice was that this headset has built-in eye tracking and pupillometry sensors. However, any VR goggles that include these features can serve as an alternative. Additionally, the HP Reverb G2 Omnicept goggles have high-quality 2160x2160 LCD panels per eye. The interpupillary distance (IPD) can be adjusted and four cameras are built into the headset to track the movement of the controllers.

### Results

**Figure 3:**
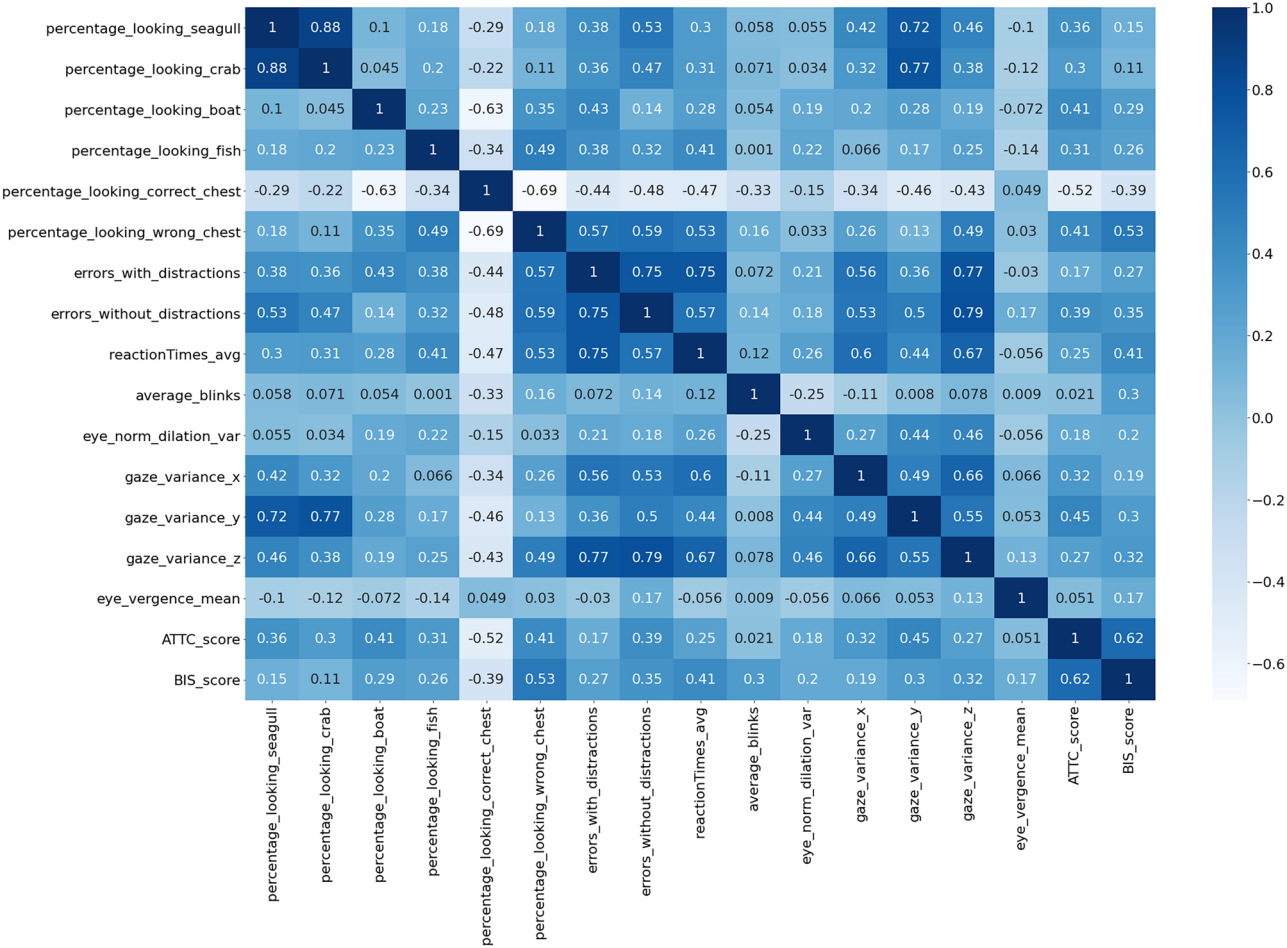
Correlation matrix between the predictors and the ATTC and BIS-11 scale scores.

Before moving on to the results of the repeated validation experiment described above, and in order to obtain a first insight, we present the correlation matrix between the predictors and the scores of attention and impulsivity (see Fig. 3).

Regarding the ATTC score, the highest absolute correlation value can be observed in the time looking at the correct chest. The value -0.52 indicates that the relationship is inverse, the longer players are able to keep their attention on the correct chest, the lower the ATTC score (the more attentive). There were also high positive correlations between ATTC score and the time spent looking at the distractors (boat 0.41, seagulls 0.36, fish 0.31, and crabs 0.3). This shows that the higher the ATTC score (more inattentive), the more time spent looking at the distractors, or at the wrong chest (correlation 0.41). We observe a correlation of 0.39 in the errors made in those sequences that did not have distractors. Interestingly, looking at the eye gaze correlation values (x 0.32, y 0.45, z 0.27), there is a positive correlation between the variability of eye movement in those directions and the ATTC score. Additionally, the eye dilation correlation is 0.18.

In terms of impulsivity, we see a correlation of 0.53 between a BIS-11 score with the time spent looking at the wrong chest. Besides, the correlation between the time looking at the correct chest and the BIS-11 score is inverse −0.39 (less impulsive subjects tend to look more to the correct chest). Errors made with or without distractors have a positive correlation with impulsivity (0.27 and 0.35 respectively). Like it happened with attention, there is a positive correlation between eye movement (x 0.19, y 0.3, z 0.32). Pupil dilation, eye vergence and blinks also show a positive correlation with impulsivity (0.2, 0.17 and 0.3, respectively). A positive correlation between impulsivity and inattention with respect to reaction time (0.41 and 0.25 respectively) was also found.

Next, we present the model’s predictive performance. In order to obtain a reference baseline, the average of the target scores of the training group was used as an estimate of the participants’ scores. The results are summarized in Table 1. Regarding the results for attention, the baseline RMSE was 8.946, whereas the test RSME was 8.269. The test correlation was 0.434. Regarding the results for impulsivity, the baseline RMSE (11.203) was again higher than the test RSME (10.538). The test correlation was 0.382.

**Table 1:** RMSE and correlations obtained by the model for attention and impulsivity. The test RMSE was better than the baseline for both constructs .

	Attention	Impulsivity
Baseline RMSE	8.946	11.203
Test RMSE	8.269	10.538
Test correlation	0.434	0.382

**Figure 4:**
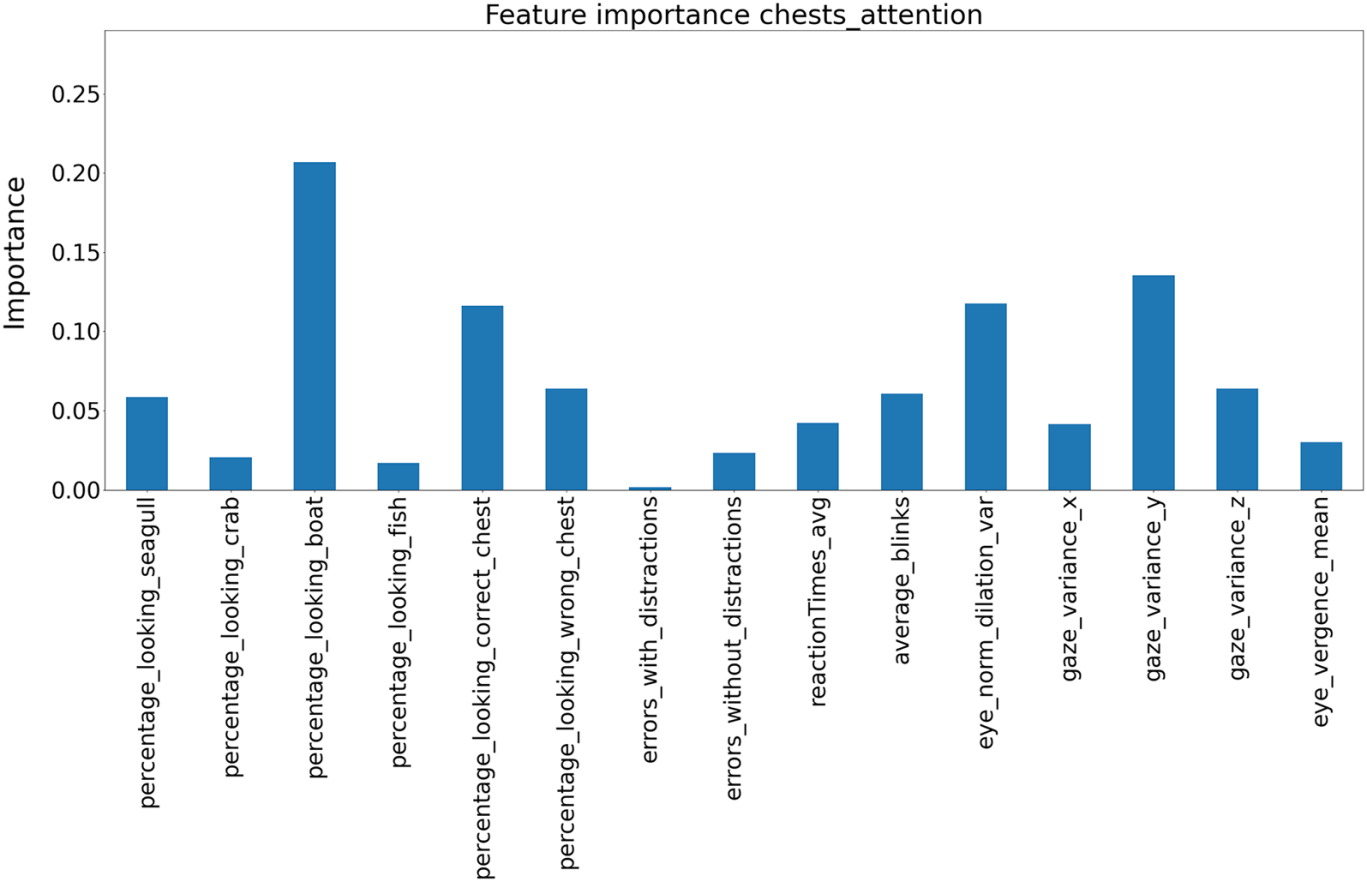
Features importance between the chest game and the attention score.

**Figure 5:**
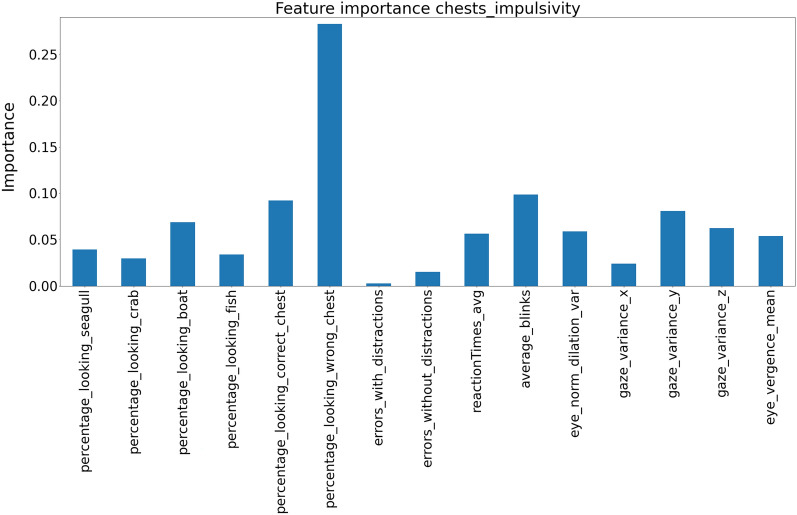
Features importance between the chest game and the impulsivity score.

The models also output the features’ importance; this can be understood as the weight each model gives to the variables. In the case of attention (see Fig. 4), the model indicates as the most important variables the time spent looking at the boat (distractor), together with the eye movements variability in the vertical axis. Regarding the most important variables for predicting impulsivity according to the model (see Fig. 5), the time looking at an incorrect chest was the most important variable and then the number of blinks.

In the next section, we will discuss these results and compare them with previous results in the literature.

### Institutional review

The study was conducted in accordance with the Declaration of Helsinki, and approved by the Ethics Committee of Universidad Autónoma de Madrid (protocol code CEI-125-2575 and date of approval 15/07/2022).

### Informed consent

Informed consent was obtained from all subjects involved in the study.

## Discussion

In this work, we have proposed the Treasure Hunt VR application that gathers performance and behavioural data that are then used to accurately predict the scores of the ATTC and BIS-11 self-report scales. One of the advantages of the model is that it allows us to know which are the most informative variables (see Figs. 4 and 5). Our results are generally consistent with what multiple studies suggest as important regressors for assessing attention and impulsivity.

First of all, errors are the most common predictors in the literature^43^. Both high impulsivity and inattention make the participant more likely to make mistakes while performing a task. Previous studies showed that there is a clear relationship between the number of errors and the level of inattention and impulsivity^21,40,44^. The results of our model corroborate this.

Also of interest are the results related to the distractors. For example, some studies claim that distractors help to assess ADHD^19,45^. In our case, there is a positive correlation between the percentage of time looking at the distractors and the test scores. This correlation is higher with the attention score than with the impulsivity score. It should be noted that the different distractors have different influences on the user. The most relevant distractor seems to be the boat, which is also the biggest. In comparison, the crab distractor, perhaps because it is smaller or because its movement is limited to a single small area, has less influence.

Another interesting variable is the reaction time. In the literature, we can find that reaction time can be a predictor of the user’s attention^44^. An inattentive person will take longer to react, and an impulsive person will have more variability in reaction time^38^. In our case, although the reaction time is not the most important variable according to the model, we can observe that it correlates positively with ATTC and BIS-11 scores. This could be due to the fact that players who had followed the impulse to look at the distractors, at the moment when the movement of the chests stopped, could take longer to choose the treasure chest.

Our model shows that eye predictors are also related to inattention and impulsivity. According to our results, it is remarkable the positive correlation between eye vergence with impulsivity, agreeing with the results of Varela et al.^36^. Our results show the importance of the number of blinks for impulsivity assessment. This is consistent with the work of Ray Li et al.^46^ where they detected a greater number of blinks in participants with higher impulsivity scores. Also, our results show that gaze variance is related to both inattentiveness and impulsiveness. Lev et al.^47^ found that ADHD patients spent more time gazing at irrelevant regions.

Furthermore, our model shows that the variance in pupil dilation is an important variable. However, the relationship is direct (more inattention, more dilation variability). This result seems at odds with some others in the literature^48,49^ but this can be due to the fact that the task in those studies was different since it was a visuospatial working memory task.

It has been observed that it is possible to obtain information about the player in an objective way, so that it can be used to predict the degree of attention and impulsivity. This is relevant because it minimizes the problem of subjectivity or malingering when answering scales or questionnaires. Our proposal differs from previous works because it has a game-like appearance and uses virtual reality devices, which allows us to continuously collect a lot of information to get a more global view of player behaviour. In addition, rather than looking at the number of errors made while distractors are present, as most studies do, we analyze the entire time spent looking at the distractors. These findings contribute to the field by highlighting the importance of registering temporal information, instead of simply using, for instance, the number of errors. Additionally, this work shows that it is possible to use machine learning techniques to help in the analysis and prediction of constructs, in this case, the degree of attention and impulsivity. The potential of these techniques could also be used to help validate new scales or questionnaires that measure the same constructs.

Our approach sheds light on which of the variables collected are more relevant and correlate more with the score on the scales measuring the degree of impulsivity and inattention. Additionally, it opens the field to other studies that could focus on predicting other constructs. However, we recommend developers of therapeutic, diagnostic or assessment games to be very specific about which constructs or executive functions they wish to measure or improve^18^. In this way, the game can be designed as simply as possible to focus only on the target constructs or executive functions to minimize interference of other constructs or EFs.

In general, the analysis of the data shows that our results are consistent with previous findings in the literature, which suggests that using VR applications including games like the one presented can be useful as an additional way of information to assess attention and impulsivity.

### Limitations

The main limitation of our work is the small sample size which may not be enough to obtain a better generalization of the model. Another limitation is that occlusions when registering hands movements with this particular version of the hardware led us to discard the hands’ data for the analysis. Our study was also limited by the variables the VR device was able to register. As discussed in the related work, other devices could provide new information that could be included to improve the predictions. Additionally, some subjects in our study suggested that the duration of 10 minutes per game was too long and that they might feel somewhat overwhelmed. Further work should study the optimal duration of the games depending on the target sample.

## Conclusion

In this work, we developed a VR application including a game to capture variables that were then used to create a machine learning model to predict the level of attention and impulsivity of the players.

A theoretical contribution of our work is that we have shown the usefulness of recording objective variables continuously. This allows us to study user behavior, seeing not only the things the user has done, but also observing the time spent on each action. Our method provides a way of knowing which variables are the most important when generating a predictive model for inattention and impulsivity and we plan to study how this could assist in the diagnosis of ADHD in future work. The random forest model can help us to improve the effectiveness of new VR applications since it can tell which variables or game mechanics are less relevant and therefore susceptible of being eliminated. For example, in our case, the model indicates that the distractor that affects the least is the crab.

Finally, the hardware currently used (HP Reverb G2 Omnicept and Laptop with an NVidia 3060 Graphics card) is relatively expensive, somewhat heavy and, the version used in this study suffered from hand capture occlusions. For future studies, we recommend the most recent version of the hardware which incorporates two additional cameras to track the hands and hence palliates this problem. Also, those kinds of VR devices may not be suitable for all individuals, which would make it difficult to apply, for the time being, the proposed approach as a general assessment technique. Hopefully, as this technology is in a maturing phase, better, cheaper and lighter solutions will become available to the common user in the coming years, enabling this type of VR application to become more widely used. Other forms of interaction could also be used in future work. For example, other motion capture devices also record the movement of other parts of the body or even brain-computer interactions.

### Supplementary Information

Supplementary Information.

## Data Availability

The datasets used and/or analysed during the current study are available from the corresponding author on reasonable request.
