# Supplementary material for: Attention and impulsivity assessment using virtual reality games
Source: Sci Rep. 2023 Aug 22;13:13689. doi: 10.1038/s41598-023-40455-4 (PMC10444747; doi:10.1038/s41598-023-40455-4)
Supplement: Supplementary file 1 — Supplementary Information. [file 41598_2023_40455_MOESM1_ESM.pdf]

Supplementary Material

Sequence	Nº movements	Speed (Units/second)	Interstimulus time (Seconds)	Has Distractors
**1**	6	3	1	No
**2**	10	4	2	No
**3**	15	4	2	No
**4**	20	4	5	Yes
**5**	20	5	2	No
**6**	30	5	10	Yes
**7**	30	6	5	No
**8**	30	6	20	No
**9**	30	7	1	Yes
**10**	40	7	10	No
**11**	40	8	1	Yes
**12**	5	8	5	No
**13**	20	5	5	No
**14**	20	5	2	Yes
**15**	30	5	10	No
**16**	30	6	15	Yes
**17**	30	6	5	Yes
**18**	30	7	1	No
**19**	40	7	10	Yes
**20**	40	8	1	No

Figure S1. Table with the characteristics of each of the 20 rounds of the defined sequence: number of chest movements, speed of the chest movements (measured in units of Unity distance per second), time set for the interval between each round and whether or not distractors appeared in a given round.
